# Mitoxantrone modulates a heparan sulfate-spike complex to inhibit SARS-CoV-2 infection

**DOI:** 10.1038/s41598-022-10293-x

**Published:** 2022-04-15

**Authors:** Qi Zhang, Peter Radvak, Juhyung Lee, Yue Xu, Vivian Cao-Dao, Miao Xu, Wei Zheng, Catherine Z. Chen, Hang Xie, Yihong Ye

**Affiliations:** 1 Laboratory of Molecular Biology, National Institute of Diabetes and Digestive and Kidney Diseases, National Institutes of Health, Bethesda, MD 20892 USA; 2 Laboratory of Pediatric and Respiratory Viral Diseases, Division of Viral Products, Office of Vaccines Research and Review, Center for Biologics Evaluation and Research, United States Food and Drug Administration, Silver Spring, MD 20993 USA; 3 National Center for Advancing Translational Sciences, National Institutes of Health, Rockville, MD 20892 USA

**Keywords:** Biochemistry, Cell biology, Drug discovery

## Abstract

Spike-mediated entry of SARS-CoV-2 into human airway epithelial cells is an attractive therapeutic target for COVID-19. In addition to protein receptors, the SARS-CoV-2 spike (S) protein also interacts with heparan sulfate, a negatively charged glycosaminoglycan (GAG) attached to certain membrane proteins on the cell surface. This interaction facilitates the engagement of spike with a downstream receptor to promote viral entry. Here, we show that Mitoxantrone, an FDA-approved topoisomerase inhibitor, targets a heparan sulfate-spike complex to compromise the fusogenic function of spike in viral entry. As a single agent, Mitoxantrone inhibits the infection of an authentic SARS-CoV-2 strain in a cell-based model and in human lung EpiAirway 3D tissues. Gene expression profiling supports the plasma membrane as a major target of Mitoxantrone but also underscores an undesired activity targeting nucleosome dynamics. We propose that Mitoxantrone analogs bearing similar heparan sulfate-binding activities but with reduced affinity for DNA topoisomerases may offer an alternative therapy to overcome breakthrough infections in the post-vaccine era.

## Introduction

The ongoing COVID-19 pandemic has claimed millions of lives worldwide and significantly damaged the global economy. Even with the development of multiple vaccines, the spread of SARS-CoV-2, the virus underlying the pandemic, is not completely halted due to breakthrough infections^1–3^. As the hope for complete eradication of this devastating virus by vaccines dwindles, the call for effective therapies to save the lives of those infected, particularly the elderly and patients with pre-existing conditions has become urgent.

Once entering the human airway, SARS-CoV-2 invades the airway epithelial cells by receptor-mediated membrane fusion or endocytosis^4–7^. Recent studies have identified several receptors mediating viral attachment and entry, which include angiotensin-converting enzyme 2 (ACE2), neuropilin-1, and tyrosine-protein kinase receptor UFO (AXL)^8–11^. Among them, ACE2, as the key mediator of viral entry, has been extensively characterized^12^. The N-terminal domain of ACE2 is composed of two lobes. SARS-CoV-2 uses the receptor-binding domain (RBD) in the spike (S) protein to contact the tip of one lobe. Neuropilin-1 was suggested recently as a distinct receptor for SARS-CoV-2^9,10^. This transmembrane receptor has two CUB (Complement C1r/C1s, Uegf, Bmp1) domains, one MAM (meprin/A5-protein/PTPmu) domain, and two coagulation factor domains. It interacts with the virus through a multi-basic site after the cleavage of spike by a furin protease. The affinity of spike for neuropilin-1 in vitro is at micromolar levels, significantly weaker than its interaction with ACE2. AXL is another recently reported candidate receptor. It specifically interacts with the N-terminal domain of the S protein. Since AXL is not co-expressed with ACE2 in human lung tissues, it was suggested as an ACE2 independent receptor for SARS-CoV-2^11^.

The efficient entry of SARS-CoV-2 also requires two independent proteolytic reactions that activate the S protein. During viral assembly, the S protein is cleaved by a host furin protease, generating two segments: S1 and S2. S1 has the RBD domain mediating receptor binding^7^. S2 drives membrane fusion as it contains a fusion peptide (FP), the functional fusogenic element^13,14^, which is activated by an additional cleavage at a site near the FP in the S2 segment. Depending on the subcellular location of the fusion reaction, the second cleavage could be mediated by either a cell surface protease (e.g. TMPRSS2) or a lysosomal protease such as Cathepsin L^8,15^. In vitro, spike- and ACE2-mediated membrane fusion could even be activated by trypsin, a non-specific protease^15^. Thus, the protease sensitivity of the S2 domain is a major determinant of viral entry efficiency^4,7,16^.

In addition to above-mentioned receptors, we and others recently reported that SARS-CoV-2 also uses the cell surface heparan sulfate proteoglycans (HSPGs) as an attachment factor during cell entry^17–19^. HSPGs refers to a family of membrane proteins conjugated with negatively charged heparan sulfate^20^, which can attract a variety of cargos to the cell surface. This presumably increase the cargo dwell time, and therefore, promote cargo entry via endocytosis^18,21^. Intriguingly, heparan sulfate can bind directly to spike^18,19,22–26^, forming a ternary complex that also includes ACE2^17^. Because heparan sulfate is essential for the recruitment of viral particles to the cell surface, drugs targeting the spike-heparan sulfate complex may interfere with the SARS-CoV-2 entry process independently of viral entry receptors.

Although viral receptors are generally considered as an ideal target for antiviral drug development, the existence of redundant SARS-CoV-2 receptors has imposed a challenge for this drug development strategy^27–29^. Our recent drug repurposing screen identified an FDA-approved anti-cancer drug named Mitoxantrone, which binds heparan sulfate and the heparan sulfate analog heparin with high affinities^18^. Mitoxantrone inhibits the entry of pseudo-viral particles coated with the SARS-CoV-2 S protein, but the underlying mechanism is unclear. Importantly, whether Mitoxantrone can block the entry of authentic SARS-CoV-2 virus is unknown. In this study, we show that Mitoxantrone targets a spike-heparin/heparan sulfate complex, restricting the membrane fusion competency of spike. As a result, Mitoxantrone inhibits the entry of an authentic SARS-CoV-2 strain in human lung epithelial cells and in a human lung EpiAirway 3D tissue model. Thus, a small-molecule COVID-19 therapeutics could be potentially developed by modifying Mitoxantrone, removing the undesired topoisomerase binding activity while keeping the heparin/heparan sulfate binding moiety.

## Results

### Mitoxantrone targets a spike-heparin complex

**Figure 1:**
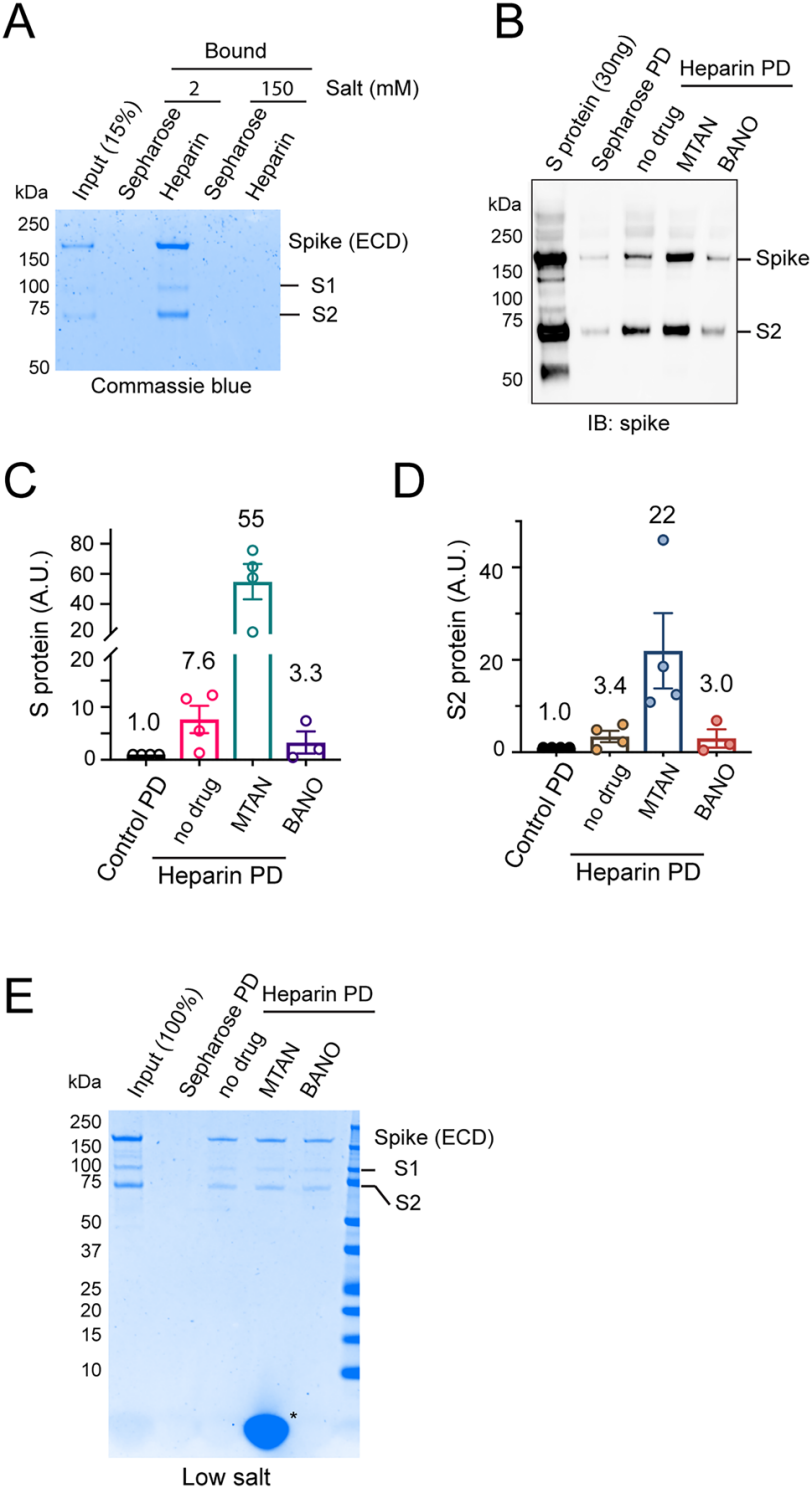
Mitoxantrone affects the spike-heparin interaction. **(A)** Spike binds to heparin in a salt sensitive manner. Purified spike ECD was incubated with either Sepharose or heparin-coated Sepharose in a buffer containing either low salt (2 mM) or 150 mM salt. Bound proteins and a fraction of input were analyzed by SDS-PAGE and Coomassie blue staining. **(B–D)** Mitoxantrone but not Banoxantrone enhances the interaction of spike with heparin. **(B)** Heparin (HP) Sepharose beads were pre-incubated with a buffer in the absence or presence of 50 μM Mitoxantrone (MTAN) or Banoxantrone (BANO). After removing unbound drugs, the beads were incubated with recombinant spike ECD. Proteins precipitated were analyzed by immunoblotting with spike antibodies. PD, pulldown. **(C,D)** Quantification of the full-length spike ECD (S) or the furin-cleaved spike (S2) in **(B)**. n = 4 independent experiments. **(E)** Mitoxantrone does not affect heparin-spike interaction under low salt conditions. As in **(B)**, except that spike was pulled down in a low salt buffer and that the samples were analyzed by Coomassie blue staining. Asterisk indicates Mitoxantrone bound to heparin beads.

Because heparin/heparan sulfate binds to both Mitoxantrone and spike, we thought that these polysaccharides might use the same binding site to interact with Mitoxantrone and spike. If so, Mitoxantrone might compete with spike for binding to HSPG on the cell surface. To test this idea, we conducted pulldown experiments by incubating the purified extracellular domain (ECD) of spike with heparin-conjugated beads in the presence of 150 mM salt or in a low salt buffer (see methods). Coomassie blue staining showed efficient co-precipitation of spike with heparin under low salt conditions, but the interaction was significantly attenuated by salt (Fig. 1A), consistent with the proposed charge-charge interactions between spike and heparin/heparan sulfate. As a control, Sepharose lacking heparin did not precipitate spike even under low salt conditions. In the presence of 150 mM salt, the interaction of spike with heparin could only be detected by immunoblotting. Interestingly, when heparin beads were first incubated with excess Mitoxantrone, prebound Mitoxantrone did not inhibit the spike-heparin interaction under this condition. Instead, Mitoxantrone enhanced spike binding to heparin by ~ 7-fold. By contrast, Banoxantrone, a compound structurally homologous to Mitoxantrone, did not show such an effect (Fig. 1B–D). Under low salt conditions, neither Mitoxantrone nor Banoxantrone affected the spike-heparin interaction (Fig. 1E). These data suggest that Mitoxantrone may change heparin (e.g. charge distribution) to increase its affinity to spike. However, our data cannot rule out the possibility that Mitoxantrone may act on spike as well.

**Figure 2:**
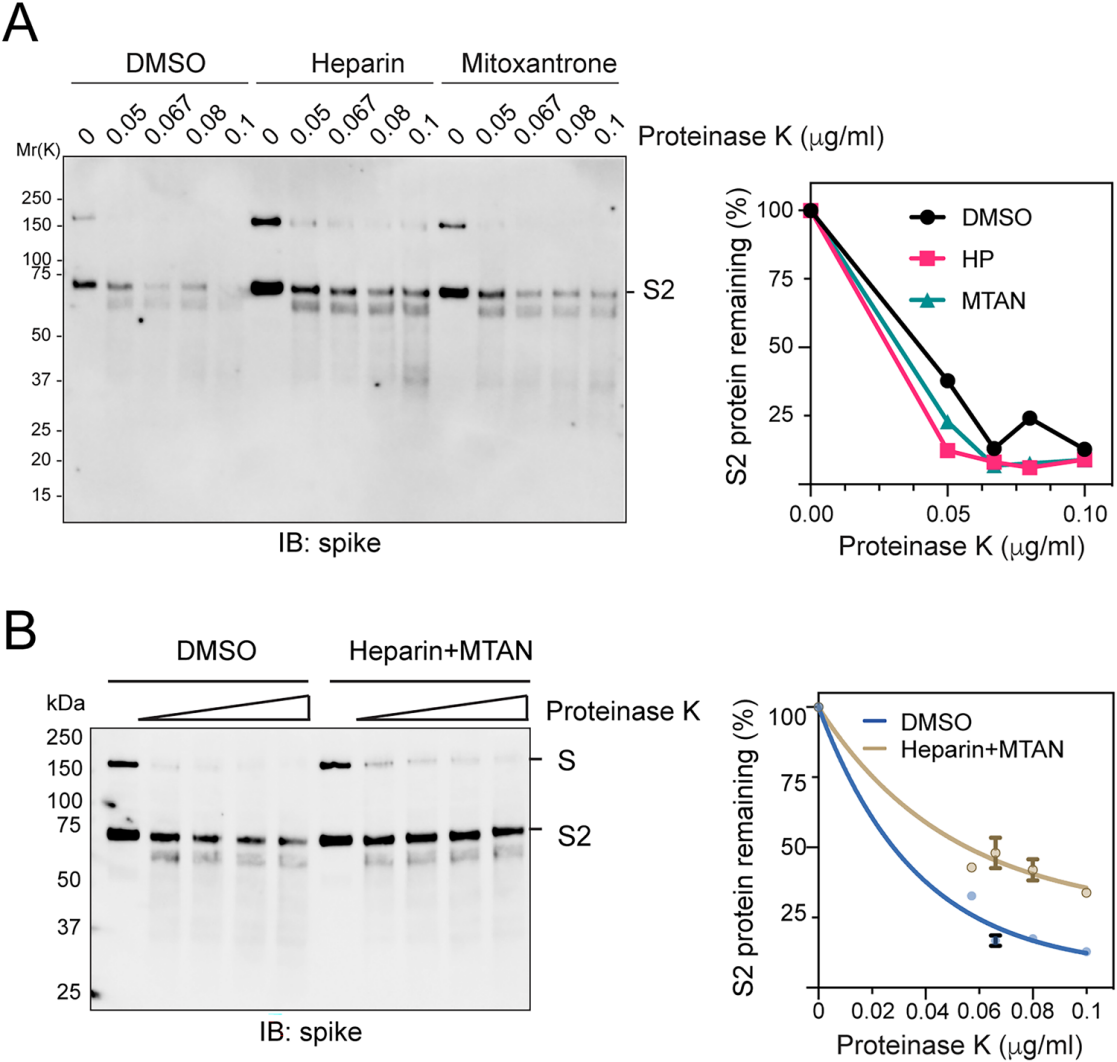
Heparin and Mitoxantrone collectively reduce protease sensitivity of spike. **(A)** Either heparin or Mitoxantrone alone does not affect the protease sensitivity of spike. Spike ECD incubated with heparin (25 μM) or Mitoxantrone (25 μM) or with a buffer control was treated with increased concentrations of proteinase K for 10 min. The samples were analyzed by immunoblotting with spike antibodies. The graph shows the quantification of the S2 band in **(A)**. **(B)** Heparin binding in the presence of Mitoxantrone increases the protease resistance of spike. As in **(A)**, except that spike was treated with heparin and Mitoxantrone together before proteolysis. The graph shows the quantification of the S2 band in **(B)**. n = 3 independent experiments. Error bars, S.E.M.

Our data raised the possibility that Mitoxantrone binding might cause a change in the conformational dynamics of the spike-heparin complex. To test this hypothesis, we treated purified spike ECD with low concentrations of proteinase K in the absence or presence of Mitoxantrone and/or heparin. Both the full-length spike ECD and the furin-processed S2 domain were readily degraded by proteinase K regardless of whether Mitoxantrone or heparin was present (Fig. 2A). However, when Mitoxantrone and heparin were both present, the S2 domain of spike was more resistant to proteolysis (Fig. 2B), suggesting that the conformational dynamics of spike may be altered by heparin and Mitoxantrone. Alternatively, the bound drug and polysaccharide may partially shield the spike S2 cleavage site.

### Mitoxantrone inhibits spike-mediated membrane fusion

**Figure 3:**
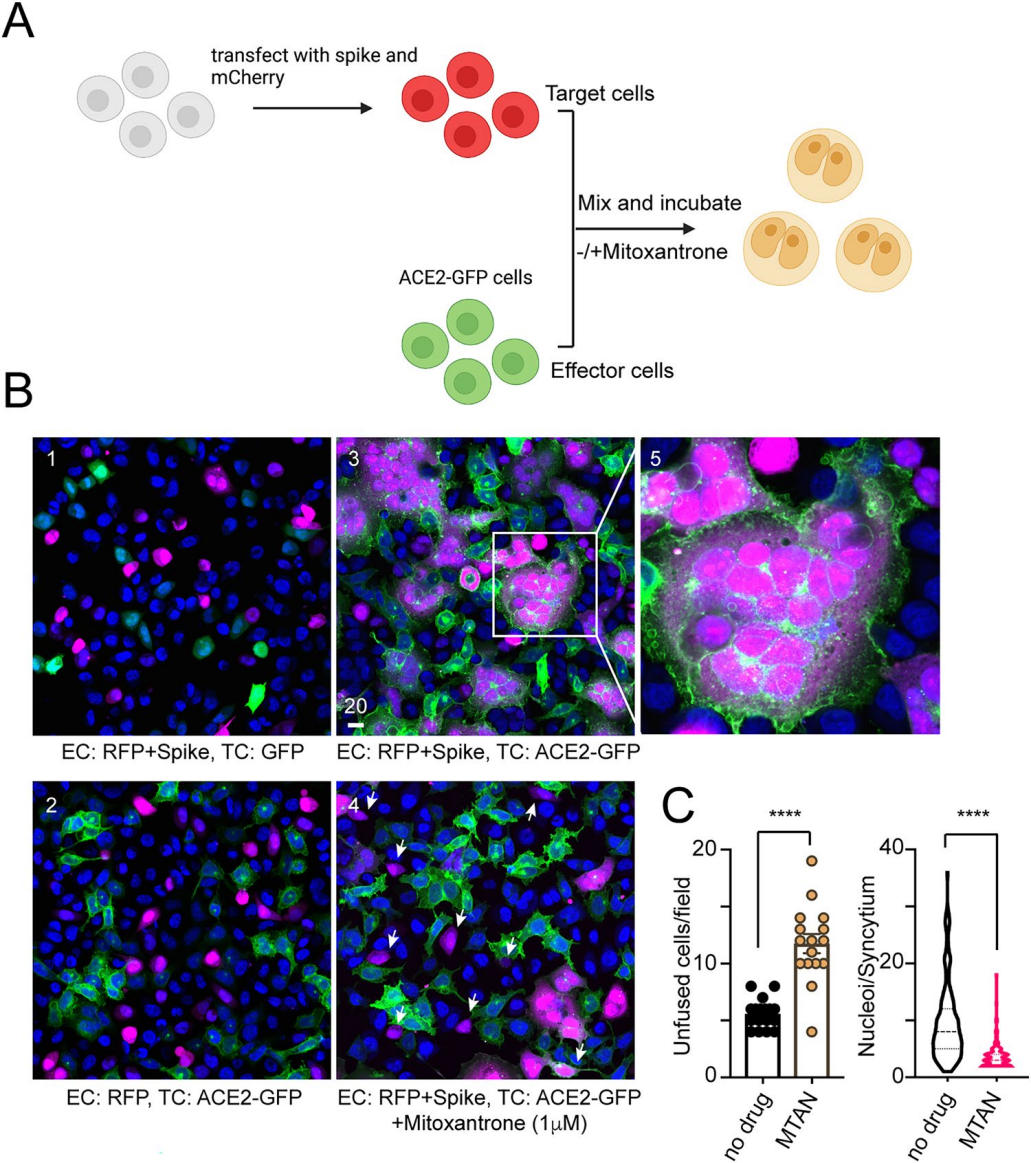
Mitoxantrone inhibits spike- and ACE2-mediated membrane fusion. **(A)** A scheme of the membrane fusion assay. (Created by Biorender.com). **(B)** Mitoxantrone inhibits spike and ACE2-mediated membrane fusion. Effect cells (EC) and target cells (TC) transfected with the indicated plasmids were mixed in equal numbers and cultured in the absence (panels 1–3) or presence (panel 4) of Mitoxantrone (1 μM) for 90 min. Cells were fixed and imaged by confocal microscopy. Panel 5 shows an enlarged view of the boxed area in panel 3. Arrows in panel 4 show example of unfused target cells. Scale bar, 20 μm. **(C)** Quantification of the experiments shown in **(B)**. The left panel shows the number of unfused cells/field, while the right panel shows the number of nuclei/syncytium. ****, p < 0.0001 by unpaired student’s t-test. n = 2 independent experiments.

The reduced sensitivity of the S2 domain to protease by heparin and Mitoxantrone treatment raised the possibility that Mitoxantrone may block spike-mediated membrane fusion. To test this, we established an in vitro membrane fusion assay by mixing two populations of HEK293T cells expressing spike and ACE2-GFP, respectively (Fig. 3A). To identify spike-positive cells, the spike-expressing plasmid was co-transfected with a mCherry-expressing plasmid. When mCherry and spike co-transfected cells were incubated with GFP-transfected cells or when mCherry-expressing cells were mixed with ACE2-GFP positive cells, no cell fusion was observed as green and red cells were segregated (Fig. 3B, panels 1, 2). However, when spike-, mCherry-positive cells are incubated with ACE2-GFP cells at 1:1 ratio for 90 min, we observed rapid cell–cell fusion, resulting in the mixing of green and red fluorescence in giant syncytia each filled with multiple nuclei (panels 3, 5). Interestingly, when cell fusion was carried out in the presence of Mitoxantrone, the cell–cell fusion was significantly inhibited as indicated by increased number of unfused cells or reduced syncytium size (panel 4, quantification in Fig. 3C). Altogether, we concluded that Mitoxantrone modulates a heparan sulfate-spike complex, changing the protease sensitivity of the S2 domain, which inhibits spike-mediated membrane fusion.

### Mitoxantrone inhibits the cell entry of an authentic SARS-CoV-2 strain

**Figure 4:**
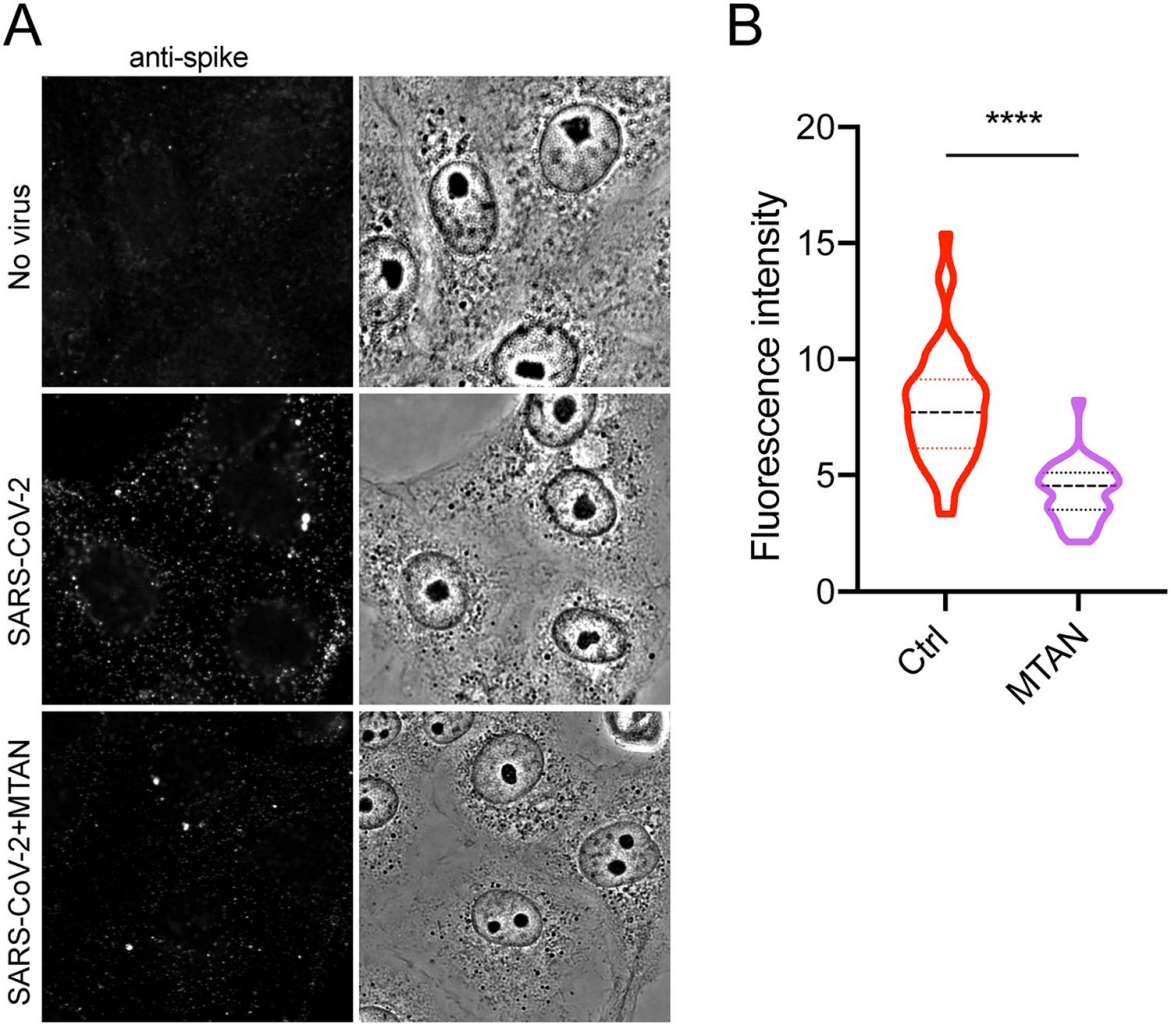
Mitoxantrone inhibits the entry of SARS-CoV-2 into a lung epithelial cell line. **(A)** Cells grown in monolayer were treated with Mitoxantrone (200 nM) and then infected with SARS-CoV-2 (USA-WA1/2020) at an MOI of 0.1. Five hours later, cells were fixed and stained with rabbit anti-spike antibodies in combination with goat anti-rabbit IgG conjugated with Alexa Fluor 488 (left panels). **(B)** Quantification of spike fluorescence intensity in **(A)**. ****, p < 0.0001 by unpaired student’s t-test, n = 3 independent experiments.

To examine whether Mitoxantrone could block the entry of an authentic SARS-CoV-2 strain, we treated cells with Mitoxantrone or as a control, with DMSO, and then exposed these cells to SARS2-CoV-2 (USA-WA1/2020) at an MOI of 0.1 for 5 h. We then stained the cells with an antibody specific for the S protein to detect viral particles inside the cells. To demonstrate the antibody specificity, uninfected cells were stained in parallel, which showed only a low diffusive background signal (Fig. 4A, top panels). By contrast, control cells infected with SARS-CoV-2 contained many bright spike-positive puncta throughout the cytoplasm (Fig. 4A, middle panels). Strikingly, when Mitoxantrone-treated cells were infected and stained, we detected a significantly lower number of spike-positive signals compared to DMSO-treated cells (Fig. 4A, bottom panels; Fig. 4B). These results suggest that like pseudo-viral particles, the infection of authentic SARS-CoV-2 is also inhibited by Mitoxantrone.

### Mitoxantrone inhibits SARS-CoV-2 infection in a human 3D EpiAirway model

**Figure 5:**
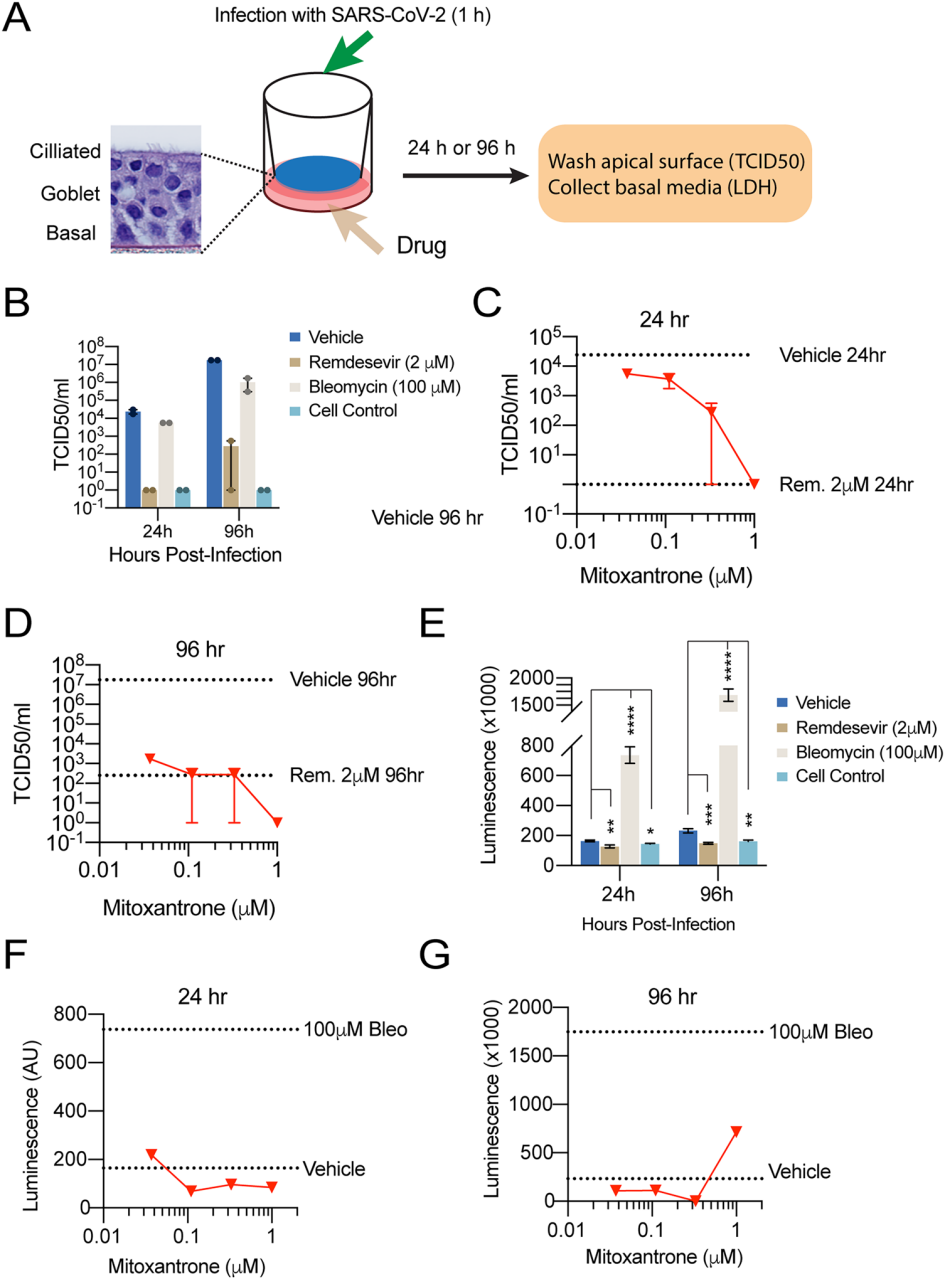
Mitoxantrone inhibits SARS-CoV-2 infection in an EpiAirway 3D tissue model. **(A)** A schematic diagram of the experimental design. **(B)** Remdesivir (2 μM) but not Bleomycin (100 μM) inhibits SARS-CoV-2 infection. 24 or 96 h after drug treatment and viral infection (MOI 0.1), the cell surface was washed. The viral titer (TCID50) in the wash was determined. **(C,D)** Mitoxantrone inhibits SARS-CoV-2 infection in the EpiAirway 3D model. TCID50 was determined either 24 h **(C)** or 96 h **(D)** after the organoids were treated with the drug at the indicated concentrations and then air-infected with SARS-CoV-2 at MOI 0.1 for 1 h. The cells were washed from the apical side to remove the virus in the cell exterior and then incubated for 24 **(C)** or 96 h **(D)**. Cells were rinsed from the apical side again and viral titers in the wash were determined. The dashed lines indicate the viral titer from cells infected without Mitoxantrone or in the presence of 2 mM Remdesivir (Rem.), as indicated. **(E)** Bleomycin (100 μM) but not Remdesivir (2 μM) induces cell death, releasing LDH as determined by a luciferase assay. *, p < 0.05, **, p < 0.01, ***, p < 0.001, ****, p < 0.0001 by unpaired student’s t-test. n = 2 tissues per test, each with 3 technical repeats. **(F,G)** Mitoxantrone inhibits SARS-CoV-2-induced cytotoxicity in the EpiAirway 3D model. As in **(D,E)** except that LDH in washes collected from Mitoxantrone-treated tissues was measured.

We next tested the anti-SARS-CoV-2 activity of Mitoxantrone in a model that mimics viral infection in the human airway. To this end, we chose human lung epithelial cell-derived 3D EpiAirway tissues, which contain three pseudostratified layers including an apical ciliated surface, the underlying mucociliary epithelium and a basal membrane. These tissues were cultured at the air–liquid interface (ALI) and can be infected with SARS-CoV-2 from the apical side (Fig. 5A)^30,31^. One hour before the infection, Mitoxantrone of different concentrations was added to the medium. After infection, uninfected viral particles were removed by gentle wash. The tissues were maintained in test article-containing medium on the basolateral side for 24 or 96 h and then were carefully washed from the apical side. The levels of infection-competent SARS-CoV-2 virions in washes were determined. For positive control, we treated tissues with Remdesivir, a nucleoside analog known to inhibit SARS-CoV-2 replication^32^. Some tissues were treated with the DNA damaging drug bleomycin as a negative control, or with dimethylsulfoxide (DMSO) as a vehicle control. As expected, neither bleomycin or DMSO had any effect on viral entry and replication. By contrast, Remdesivir treatment dramatically reduced the viral load in washes collected at 24 or 96 h post-infection (Fig. 5B), confirming the anti-SARS-CoV-2 activity of Remdesivir. Consistent with our observation in cultured cells, Mitoxantrone also dose-dependently reduced the viral load in washes at both 24 and 96 h post-infection (Fig. 5C,D).

To evaluate the cytotoxicity of Mitoxantrone, we measured the level of lactate dehydrogenase (LDH) in the basal culture media. LDH is a cytosolic enzyme released to the cell exterior upon cell death. Accordingly, we detected high levels of LDH in the basal media from tissues treated with the apoptosis-inducing drug bleomycin (Fig. 5E). We also observed a small increase in LDH in the basal media collected from SARS-CoV-2-infected cells compared to uninfected control, which was prevented if cells were pretreated with Remdesivir (Fig. 5E). Virus-induced cytotoxicity and LDH release was also inhibited in Mitoxantrone-treated tissues at 24 h (Fig. 5F). At 96 h post-infection, a low concentration of Mitoxantrone continued to lower the LDH level in the basal media compared to DMSO-treated control (Fig. 5G). However, we observed a small increase in LDH level in 1 μM Mitoxantrone-treated samples (Fig. 5G). This is probably due to Mitoxantrone’s intrinsic cytotoxicity (see below). Collectively, these data suggest that Mitoxantrone has a potent anti-SARS-CoV-2 activity at concentrations with low cytotoxicity.

### Gene expression profiling reveals an undesired activity of Mitoxantrone

**Figure 6:**
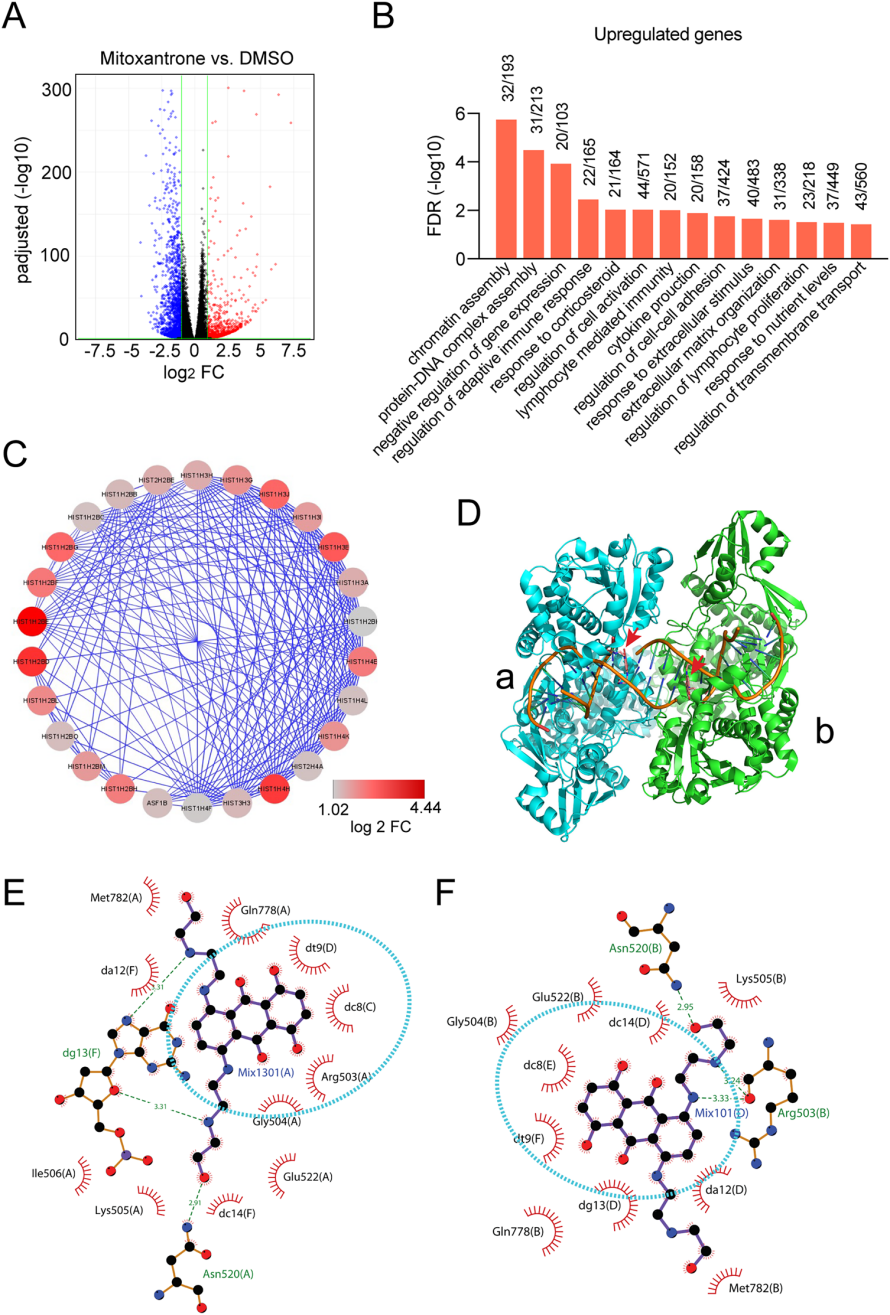
Gene expression profiling shows two major cellular targets of Mitoxantrone. **(A)** A volcano plot showing genes upregulated or downregulated by Mitoxantrone in HEK293T cells. **(B)** A list of significant pathways upregulated by Mitoxantrone. **(C)** Genes encoding nucleosome proteins are highly upregulated by Mitoxantrone. Shown is a gene interaction network highlighting nucleosome assembly-related genes induced by Mitoxantrone. Colors indicate fold change. **(D)** A crystal structure shows the binding of Mitoxantrone to a DNA-topoisomerase complex. Arrows indicate the two Mitoxantrone molecules each associated with a topoisomerase monomer. **(E,F)** LigPlot views of the molecular interactions between Mitoxantrone and the DNA-topoisomerase complex.

To understand the global impact of Mitoxantrone treatment on cells and to identify target inhibition that is irrelevant to viral entry, we analyzed the mRNA sequencing profile of HEK293T cells exposed to Mitoxantrone. This treatment dramatically changed the expression of many genes (Fig. 6A). Gene ontology (GO) analysis of the 828 genes upregulated by Mitoxantrone by at least twofold with adjusted p-values less than 0.05 in triplicated experiments showed that the enriched processes fall into two categories: they are either associated with nucleosome assembly or disassembly or linked to events at the plasma membranes such as cellular response to stimulus, response to nutrient levels, regulation of cell–cell adhesion, etc. (Fig. 6B). The latter is consistent with the observed interaction of Mitoxantrone with the cell surface HSPG, which presumably induces compensatory gene expression changes to maintain proper cellular responses to extracellular stimuli. On the other hand, the upregulation of histone genes and genes regulating nucleosome assembly (Fig. 6C) is consistent with the reported interaction of Mitoxantrone with nucleic acid and DNA topoisomerase (Fig. 6D), which is thought to account for the cytotoxicity of Mitoxantrone in cancer therapy. Structural analyses show that the di-hydroxy-anthraquinone moiety of Mitoxantrone forms extensive hydrophobic interactions with the DNA/topoisomerase complex (Fig. 6E,F). Since the anti-SARS-CoV-2 activity has been linked to the two symmetric arms, we proposed that modification of the anthraquinone moiety may reduce cytotoxicity while maintaining the heparan sulfate-binding and thus the anti-SARS-CoV-2 function of Mitoxantrone.

## Discussion

The rapid development and deployment of COVID-19 vaccines have significantly slowed down the current pandemic. Nevertheless, the evolution of new viral strains combined with the disparity in global vaccine distribution has led to new waves of infected cases and deaths. Thus, an effective and economical therapeutic agent is urgently needed, particularly in developing countries that have limited access to vaccines. Our study suggests that Mitoxantrone derivatives with reduced affinity to DNA-topoisomerase complexes may be promising anti-COVID drug candidates.

Mitoxantrone has been approved for treating acute myeloid lymphoma, prostate cancer, and multiple sclerosis. The anti-cancer activity of Mitoxantrone has been attributed to its interactions with DNA-topoisomerase complexes, which inhibit DNA replication^33^. Mitoxantrone also effectively inhibits the entry of spike-pseudotyped viral particles^18^ and authentic SARS-CoV-2 virions (this study) but the underlying mechanism has been unclear. Our study showed that Mitoxantrone has a high affinity for heparin/heparan sulfate, but it does not prevent the association of heparan sulfate with spike. Instead, it stabilizes spike in a complex with heparan sulfate, increasing its resistance to protease digestion. The presence of Mitoxantrone in a heparan sulfate-spike complex appears to reduce the fusogenic activity of spike, which in turn diminishes the infectivity of SARS-CoV-2. However, the precise mechanism by which Mitoxantrone inhibits spike-mediated membrane fusion remains to be elucidated. The binding of Mitoxantrone to heparan sulfate also diminishes the endocytosis of SARS-CoV-2 viral particles and many other HSPG cargos^18,21^. Whether these two activities of Mitoxantrone share a common mechanism is also unclear. Because Mitoxantrone targets both DNA topoisomerases and heparan sulfate in mammalian cells, a potential strategy for developing Mitoxantrone into an antiviral agent is to generate derivatives that only bind to heparan sulfate.

Compared to other viral entry inhibitors under development, Mitoxantrone is unique as it is a small molecule targeting a host factor required for viral entry. Thus, it has several advantages. For example, several neutralizing antibodies have been approved for use in patients with mild COVID symptoms. Many studies also reported promising anti-SARS-CoV-2 activities for nanobodies against spike in animal models^34–38^. Although antibody-based therapies are generally safe and effective, they require high doses, which makes these therapies quite expensive. Additionally, most neutralizing antibodies recognize the RBD of spike, which is rapidly evolving as the virus continues to spread. Consequently, new variants with mutated spike may not be recognized by existing antibodies. In this regard, a safe, small molecule inhibitor targeting a host factor required for viral entry may offer an alternative therapeutic option that can be used either as a single agent or in combination with spike antibodies or other anti-viral agents.

Drugs targeting host factors have been successfully developed to treat other infectious diseases. For example, Maraviroc, an anti-HIV agent targeting the host membrane protein CCR5 has been approved for AIDS treatment^39^. Therefore, it is tempting to generate drugs with similar heparan sulfate-binding activities as Mitoxantrone but improved safety profile. Our structural analyses suggest that the two hydroxyl groups in the anthraquinone moiety of Mitoxantrone may be the best place to introduce modifications, which are expected to reduce its binding to DNA-topoisomerase. Given the broad role of the cell surface HSPG in pathogen entry, Mitoxantrone derivatives may even be used to treat a new viral infection that exploits the cell surface HSPG for viral attachment and entry.

## Materials and methods

### Reagents and plasmids

Spike protein was purchased from Sino Biologicals. Mitoxantrone was purchased from Microsource (Cat #01503278) and stored by the NCATS compound management department. Banoxantrone was purchased from Sigma (Catalog number: SML1854). pcDNA3.1-SARS-CoV-2-Spike plasmid was obtained from BEI resource^7^. pLV-Mcherry was obtained from Addgene (Catalog number: 36084). Hoechst 33342 staining solution and proteinase K were purchased from Thermo Fisher Scientific (Catalog number: 62249 and AM2546).

### Heparin Sepharose beads pulldown and protease K digestion

Heparin pulldown assays were performed as previously reported^18^. Briefly, spike 800 ng was incubated with heparin or control Sepharose beads in a buffer (25 mM Tris 7.4, 2 mM magnesium chloride, 2 mM potassium chloride, 0.05% NP40) containing either no additional salt or 150 mM sodium chloride. After incubation at 4° for 20 min, bound materials were collected by centrifugation. After two quick washing with the binding buffer, bound proteins were eluted with the Laemmli buffer. Where indicated, heparin beads were first treated with 50 µM Mitoxantrone to saturate all binding sites before being incubated with spike.

For protease treatment experiment, 1.28 μg spike protein was incubated with either DMSO, Mitoxantrone (25 μM), heparin (25 μM) or Mitoxantrone together with heparin in the presence of 0.1 M of Tris–HCl (pH7.4) in pure water (220 μl of the total volume). Mitoxantrone and heparin were pre-mixed before adding to the S protein to avoid unwanted precipitations. After incubation at room temperature for 15 min, the reactants were aliquoted into 40 μl per tube. 10 μl pre-diluted proteinase K was added to make the final concentrations as 0.05, 0.067, 0.08, or 0.1 μg/ml. The reactions were further incubated for 5 min at room temperature and then quenched by pre-heated 4 × Laemmli buffer and heating. The samples were resolved by SDS-PAGE electrophoresis followed by immunoblotting.

### Spike- and ACE2-mediated cell fusion assay

HEK293T target cells expressing spike protein and mCherry were generated by transfecting cells with pcDNA3.1-SARS-CoV-2-Spike and pLV-Mcherry at 1:1 ratio. Effect cells were HEK293T cells stably expressing ACE2-GFP, as described previously^18^, or as a control, HEK293T cells transfected with pEGFP. To initiate cell–cell fusion, effect cells and target cells were mixed at 1:1 ratio in a live cell imaging chamber and placed in the incubator for 90 min. Cells were then fixed by 4% paraformaldehyde in phosphate buffer saline and then stained with a Hoechst 33342 staining solution to label nuclei. Cell were imaged by a Zeiss LSM780 confocal microscopy.

### SARS-CoV-2 infection on cells

7 × 10^4^ Vero E6 cells (ATCC #CRL-1586) were seeded into an Millicell EZ SLIDES chamber slides (Millipore) 24 h before infection. On the day of infection, cells were pre-treated with 200 nM mitoxantrone or as a control with DMSO for 30 min. After 30 min treatment, Vero E6 cells were infected with live SARS-Cov-2 (USA-WA1/2020) (ATCC #NR-52281) at an MOI of 0.1 for 5 h at 37 °C, 5% CO2. Cells were then washed, fixed with 4% paraformaldehyde. The viral spike protein was stained with an in-house developed rabbit anti-spike antibody and goat anti-rabbit IgG (H + L) highly cross-adsorbed antibody with Alexa Fluor 488 (Invitrogen). Images were acquired using FluoView FV10i Confocal Laser Scanning Microscope (Olympus). All live virus infections were performed in a biosafety level 3 (BSL3) laboratory as approved by the Institutional Biosafety Committee (IBC).

### SARS-CoV-2 infection in a 3D EpiAirway model

Infection in the 3D EpiAirway model was carried out by University of Louisiana as a contracted service. In general, human tracheobronchial epithelial cells (EpiAirway™ from MatTek) were culture on inserts at ALI in 6-well plates. Prior to addition of drugs or virus, accumulated mucus from the tissue surface were removed by gently rinsing the apical surface twice with 400 μl TEER buffer. All fluids from the tissue surface were carefully removed to leave the apical surface exposed to the air. Mitoxantrone and control compounds were diluted into the assay medium (AIR-ASY-100). Tissues were pretreated with compounds for 1 h on the apical and basal sides. After compound pretreatment, liquid was removed from the apical side, and virus (2019-nCoV/USA-WA1/2020 at MOI of 0.1) was inoculated in 0.15 mL assay medium onto the apical layer for 1 h. Following 1 h inoculation, virus-containing media was removed from apical layer and the apical side was washed with 400 μl TEER buffer. The basolateral medium was replaced with fresh maintenance medium. Media on the basolateral side was replaced with fresh compound containing maintenance medium at 24, 48, and 72 h post-infection.

### TCID_50_ (50% tissue culture infectious dose) assay

Human bronchial epithelial cells (HBEC’s 3D-EpiAirway™) were seeded into culture inserts for 6-well plates one day before viral infection. Prior to addition of drugs or virus, accumulated mucus from the tissue surface were removed by gently rinsing the apical surface twice with 400 μl TEER buffer. All fluids from the tissue surface were carefully removed to leave the apical surface exposed to the air. Mitoxantrone was diluted into the assay medium and placed at room temperature before co-treatment with virus (MOI: 0.1) onto apical layer and basal layer for 1 h. Following 1 h treatment, virus was removed from apical layer and basolateral medium was replaced with fresh maintenance medium and compound at 24 h, 48 h and 72 h post-infection.

At 24 h and 96 h post-infection, the apical layer was washed with 0.4 mL of TEER buffer (PBS with Mg^2+^ and Ca^2+^) and aliquoted to separate microfuge tubes (1.5 mL). Eight-fold serial dilutions of apical layer supernatant sample concentrations were added to 96-well assay plates containing Vero E6 cells (20,000/well). The plates were incubated at 37 °C, 5% CO_2_ and 95% relative humidity. Following 3 days (72 ± 4 h) incubation, the plates were stained with crystal violet to measure cytopathic effect (CPE). Virus titers were calculated using the method of Reed and Muench (Reed et al., 1938). The TCID_50_ values were determined from duplicate samples.

### LDH assay

Medium from the basolateral layer of the tissue culture inserts was removed 24- and 96-h post-infection and diluted in LDH Storage Buffer as per the manufacturer’s instructions (LDH-Glo Cytotoxicity Assay, Promega). Samples (5 μl) were further diluted with LDH Buffer (95 μl) and incubated with an equal volume of LDH Detection Reagent. Luminescence was recorded after 60 min incubation at room temperature. A no cell control was included as a negative control to determine culture medium background and bleomycin included as a positive cytotoxic control.

### Gene expression and structural analyses

To study the Mitoxantrone’s effect on gene expression in HEK293T cells, we analyzed the mRNA sequencing data from our previous study, which is stored in the NCBI sequence read archive with accession ID: PRJNA645209. We used the web-based String protein interaction network program to analyze significantly up- or down-regulated genes. The identified networks were exported into Cytoscape for graph-making. The complex of Mitoxantrone with DNA-topoisomerase was analyzed by LigPlot^40^ to reveal the interaction between Mitoxantrone and the enzyme.

To determine how Mitoxantrone interacts with DNA and topoisomerase, we used LigPlot to analyze the molecular interactions present in the published structure (PDB: 4g0v)^33^.

### Image processing and statistical analyses

Confocal images were processed using the Zeiss Zen software. To measure fluorescence intensity, we used the open-source Fiji software. Images were converted to individual channels and regions of interest were drawn for measurement. Statistical analyses were performed using either Excel or GraphPad Prism 9. Data are presented as means ± SEM, which was calculated by GraphPad Prism 9. *P* values were calculated by Student’s *t*-test using Excel. Nonlinear curve fitting and IC_50_ calculation was done with GraphPad Prism 9 using the inhibitor response three variable model or the exponential decay model. Images were prepared with Adobe Photoshop and assembled in Adobe Illustrator. Data processing and reporting are adherent to the community standards. Uncropped gel and immunoblot images are shown in supplementary Figs. 1 and 2.

## Supplementary Information

Supplementary Figure S1.

Supplementary Figure S2.

## References

[1] Juthani PV (2021). Hospitalisation among vaccine breakthrough COVID-19 infections. Lancet Infect. Dis..

[2] Le TT, Cramer JP, Chen R, Mayhew S (2020). Evolution of the COVID-19 vaccine development landscape. Nat. Rev. Drug Discov..

[3] Haas EJ (2021). Impact and effectiveness of mRNA BNT162b2 vaccine against SARS-CoV-2 infections and COVID-19 cases, hospitalisations, and deaths following a nationwide vaccination campaign in Israel: An observational study using national surveillance data. Lancet.

[4] Shang J (2020). Cell entry mechanisms of SARS-CoV-2. Proc. Natl. Acad. Sci. U S A.

[5] Wan Y, Shang J, Graham R, Baric RS, Li F (2020). Receptor recognition by the novel coronavirus from Wuhan: An analysis based on decade-long structural studies of SARS coronavirus. J. Virol..

[6] Glebov OO (2020). Understanding SARS-CoV-2 endocytosis for COVID-19 drug repurposing. FEBS J..

[7] Walls AC (2020). Structure, function, and antigenicity of the SARS-CoV-2 spike glycoprotein. Cell.

[8] Hoffmann M (2020). SARS-CoV-2 cell entry depends on ACE2 and TMPRSS2 and is blocked by a clinically proven protease inhibitor. Cell.

[9] Cantuti-Castelvetri L (2020). Neuropilin-1 facilitates SARS-CoV-2 cell entry and infectivity. Science.

[10] Daly JL (2020). Neuropilin-1 is a host factor for SARS-CoV-2 infection. Science.

[11] Wang S (2021). AXL is a candidate receptor for SARS-CoV-2 that promotes infection of pulmonary and bronchial epithelial cells. Cell Res.

[12] Zamorano Cuervo N, Grandvaux N (2020). ACE2: Evidence of role as entry receptor for SARS-CoV-2 and implications in comorbidities. Elife.

[13] Papa G (2021). Furin cleavage of SARS-CoV-2 spike promotes but is not essential for infection and cell-cell fusion. PLoS Pathog..

[14] Xia S (2020). The role of furin cleavage site in SARS-CoV-2 spike protein-mediated membrane fusion in the presence or absence of trypsin. Signal Transduct Target Ther..

[15] Ou X (2020). Characterization of spike glycoprotein of SARS-CoV-2 on virus entry and its immune cross-reactivity with SARS-CoV. Nat. Commun..

[16] Belouzard S, Chu VC, Whittaker GR (2009). Activation of the SARS coronavirus spike protein via sequential proteolytic cleavage at two distinct sites. Proc. Natl. Acad. Sci. U S A.

[17] Clausen TM (2020). SARS-CoV-2 infection depends on cellular heparan sulfate and ACE2. Cell.

[18] Zhang Q (2020). Heparan sulfate assists SARS-CoV-2 in cell entry and can be targeted by approved drugs in vitro. Cell Discov..

[19] Yue J (2021). Heparan sulfate facilitates spike protein-mediated SARS-CoV-2 host cell invasion and contributes to increased infection of SARS-CoV-2 G614 mutant and in lung cancer. Front. Mol. Biosci..

[20] Sarrazin S, Lamanna WC, Esko JD (2011). Heparan sulfate proteoglycans. Cold Spring Harb. Perspect. Biol..

[21] Zhang Q (2020). A myosin-7B-dependent endocytosis pathway mediates cellular entry of alpha-synuclein fibrils and polycation-bearing cargos. Proc. Natl. Acad. Sci. U S A.

[22] Tiwari, V. *et al.* Preferential recognition and antagonism of SARS-CoV-2 spike glycoprotein binding to 3-O-sulfated heparan sulfate. *bioRxiv*. 10.1101/2020.10.08.331751 (2020).

[23] Liu L (2021). Heparan sulfate proteoglycans as attachment factor for SARS-CoV-2. ACS Cent. Sci..

[24] Kim SY (2020). Characterization of heparin and severe acute respiratory syndrome-related coronavirus 2 (SARS-CoV-2) spike glycoprotein binding interactions. Antiviral Res..

[25] Mycroft-West CJ (2020). Heparin inhibits cellular invasion by SARS-CoV-2: Structural dependence of the interaction of the spike S1 receptor-binding domain with heparin. Thromb. Haemost..

[26] Tandon, R. *et al.* Effective inhibition of SARS-CoV-2 entry by heparin and enoxaparin derivatives. *bioRxiv*. 10.1101/2020.06.08.140236 (2020). 10.1128/JVI.01987-20 PMC7925120 33173010

[27] Artese A (2020). Current status of antivirals and druggable targets of SARS CoV-2 and other human pathogenic coronaviruses. Drug Resist. Update.

[28] Izda V, Jeffries MA, Sawalha AH (2021). COVID-19: A review of therapeutic strategies and vaccine candidates. Clin. Immunol..

[29] Twomey JD (2020). COVID-19 update: The race to therapeutic development. Drug Resist. Update.

[30] Zarkoob, H. *et al.* Modeling SARS-CoV-2 and influenza infections and antiviral treatments in human lung epithelial tissue equivalents. *bioRxiv*. 10.1101/2021.05.11.443693 (2021). 10.1038/s42003-022-03753-7 PMC9373898 35962146

[31] Zhu N (2020). Morphogenesis and cytopathic effect of SARS-CoV-2 infection in human airway epithelial cells. Nat. Commun..

[32] Beigel JH (2020). Remdesivir for the treatment of Covid-19—Final report. N. Engl. J. Med..

[33] Wu CC, Li YC, Wang YR, Li TK, Chan NL (2013). On the structural basis and design guidelines for type II topoisomerase-targeting anticancer drugs. Nucleic Acids Res..

[34] Xu J (2021). Nanobodies from camelid mice and llamas neutralize SARS-CoV-2 variants. Nature.

[35] Ma H (2021). Potent neutralization of SARS-CoV-2 by hetero-bivalent alpaca nanobodies targeting the spike receptor-binding domain. J. Virol..

[36] Huo J (2020). Neutralizing nanobodies bind SARS-CoV-2 spike RBD and block interaction with ACE2. Nat. Struct. Mol. Biol..

[37] Koenig PA (2021). Structure-guided multivalent nanobodies block SARS-CoV-2 infection and suppress mutational escape. Science.

[38] Xiang Y (2020). Versatile and multivalent nanobodies efficiently neutralize SARS-CoV-2. Science.

[39] Sierra-Madero JG (2014). Effect of the CCR5 antagonist maraviroc on the occurrence of immune reconstitution inflammatory syndrome in HIV (CADIRIS): A double-blind, randomised, placebo-controlled trial. Lancet HIV.

[40] Wallace AC, Laskowski RA, Thornton JM (1995). LIGPLOT: A program to generate schematic diagrams of protein-ligand interactions. Protein Eng..

