# Supplementary figures and images for: Mitoxantrone modulates a heparan sulfate-spike complex to inhibit SARS-CoV-2 infection
Source: Sci Rep. 2022 Apr 15;12:6294. doi: 10.1038/s41598-022-10293-x (PMC9016215; doi:10.1038/s41598-022-10293-x)

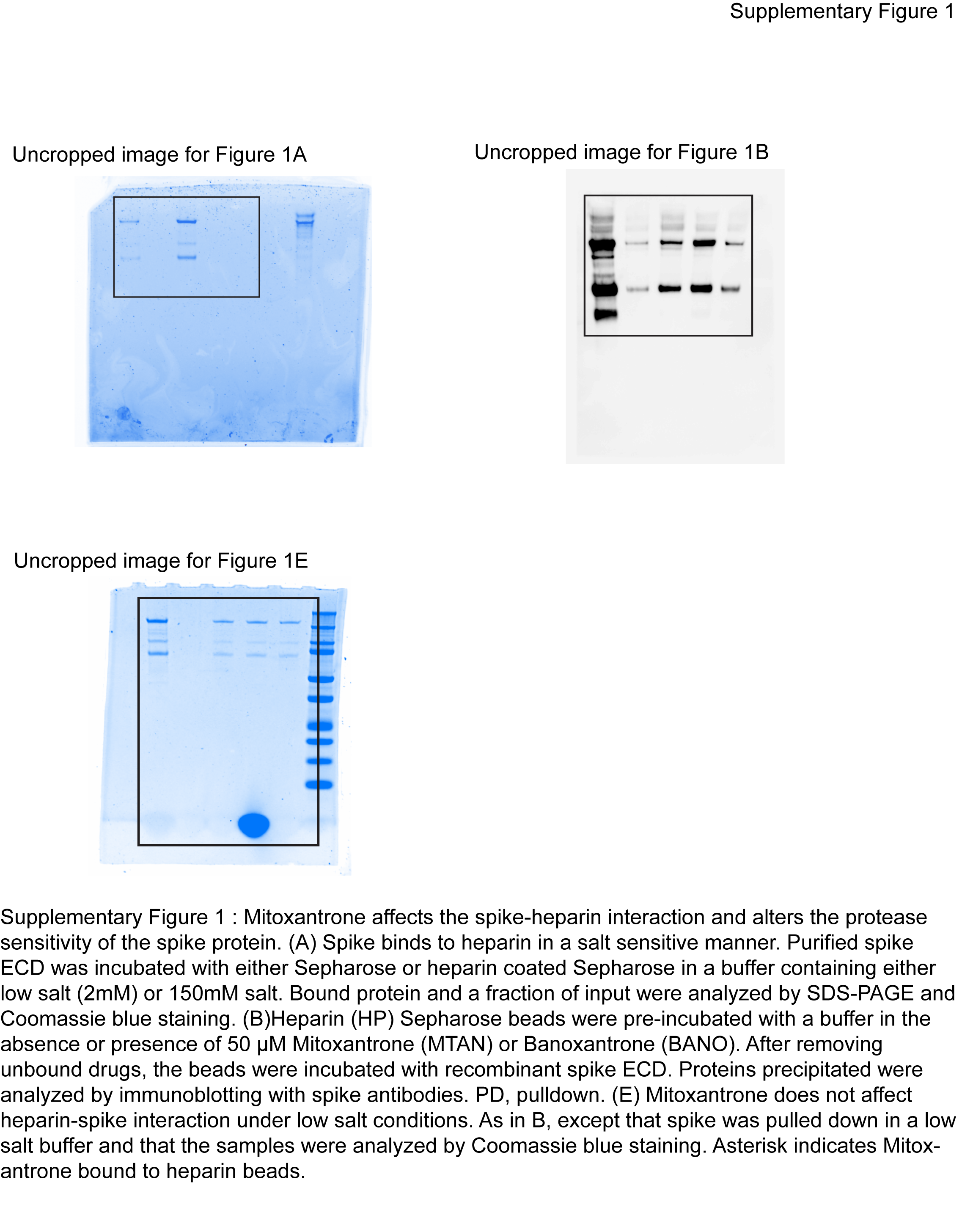

Supplement: Supplementary file 1 — Supplementary Figure S1. [file 41598_2022_10293_MOESM1_ESM.tif]

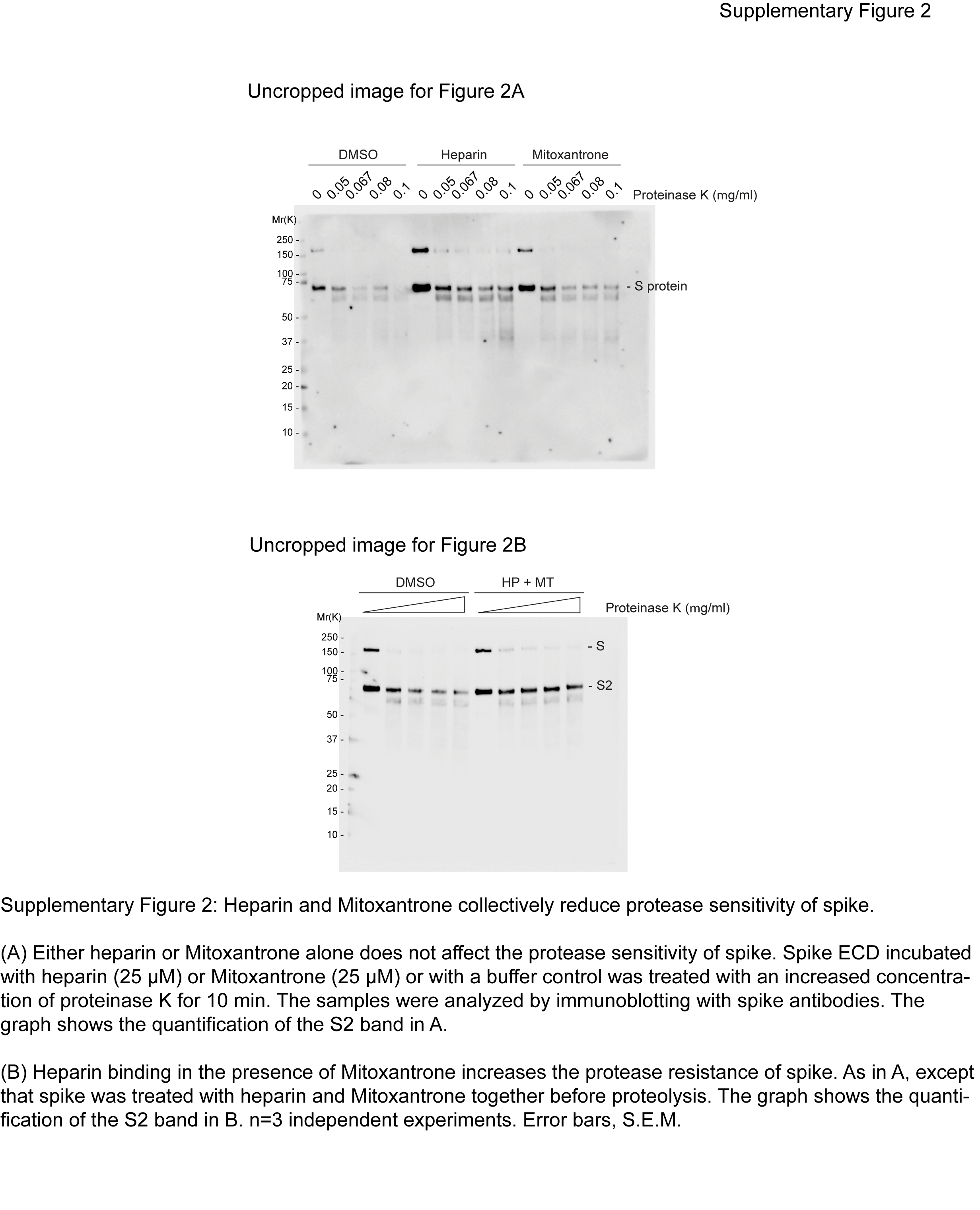

Supplement: Supplementary file 2 — Supplementary Figure S2. [file 41598_2022_10293_MOESM2_ESM.tif]
